# A participatory surveillance of marsh deer (*Blastocerus dichotomus*) morbidity and mortality in Argentina: first results

**DOI:** 10.1186/s12917-020-02533-x

**Published:** 2020-09-01

**Authors:** M. Marcela Orozco, Hernán D. Argibay, Leonardo Minatel, Eliana C. Guillemi, Yanina Berra, Andrea Schapira, Dante Di Nucci, Andrea Marcos, Fernanda Lois, Martín Falzone, Marisa D. Farber

**Affiliations:** 1 Instituto de Ecología, Genética y Evolución de Buenos Aires, IEGEBA-CONICET, Intendente Güiraldes 2160, Ciudad Universitaria, C1428EGA Ciudad Autónoma de Buenos Aires, Argentina; 2 Facultad de Ciencias Exactas y Naturales, Universidad de Buenos Aires, Intendente Güiraldes 2160, Ciudad Universitaria, C1428EGA Ciudad Autónoma de Buenos Aires, Argentina; 3 Cátedra de Patología, Facultad de Ciencias Veterinarias, Universidad de Buenos Aires, Av. San Martín 5285, C1217DSM Ciudad Autónoma de Buenos Aires, Argentina; 4 Instituto de Biotecnología-IABiMo, Instituto Nacional de Tecnología Agropecuaria (INTA- CONICET), Las Cabañas y Los Reseros s/n, B1712WAA Castelar, Argentina; 5 Área Salud Pública, Facultad de Ciencias Veterinarias, Universidad de Buenos Aires, Av. Chorroarín 280, C1427CW Ciudad Autónoma de Buenos Aires, Argentina; 6 Fundación de Historia Natural Félix de Azara, Hidalgo 767, C1405BCK Ciudad Autónoma de Buenos Aires, Argentina; 7 Servicio Nacional de Sanidad y Calidad Agroalimentaria (SENASA), Av. Paseo Colón 367, C1063ACD Ciudad Autónoma de Buenos Aires, Argentina; 8 Fundación Temaikèn, Ruta Provincial 25, B1625 Belén de Escobar, Buenos Aires, Argentina

**Keywords:** Participatory surveillance, Networks, Marsh deer, *Blastocerus dichotomus*

## Abstract

**Background:**

In an era of unprecedented socio-ecological changes, managing wildlife health demands high-quality data collection and the engagement of local communities. *Blastocerus dichotomus*, the largest South American deer, is Vulnerable to extinction mainly due to habitat loss. Diseases have been recognised as a potential threat, and winter mortality has been historically described in marsh deer populations from Argentina. Field difficulties have, however, prevented in-depth studies of their health status.

**Results:**

Between May 2014 and April 2017, we investigated marsh deer morbidity and mortality in the two largest populations in Argentina. We collected data by means of a passive surveillance system that involved a network of researchers, field partners (veterinarians, park rangers, and local community), and decision makers. We sampled marsh deer during as well as outside mortality events. A total of 44 marsh deer with different body condition scores were evaluated. We obtained haematology and biochemistry values from animals with good body condition score. Marsh deer with poor body condition had a high burden of the ticks *Amblyomma triste* and *Rhipicephalus microplus*. Vector-borne agents such as *Theileria cervi*, *Trypanosoma theileri, Trypanosoma evansi, Ehrlichia chaffeensis, Anaplasma platys, Anaplasma odocoilei, Anaplasma marginale*, and *Candidatus Anaplasma boleense* were also found. *Haemonchus* spp., *Ostertagia* spp., and *Trichostrongylus* spp. were the most frequent gastrointestinal parasites in deer with poor body condition. A Multiple Correspondence Analysis reinforced a possible association of winter period with lower body score condition, high tick loads, infection with *E. chaffeensis*, and presence of harmful gastrointestinal parasites.

**Conclusions:**

Our approach allowed the establishment of a participatory surveillance network of marsh deer morbidity and mortality in Argentina. We report and analyse the first data obtained opportunistically within the framework of this network, providing information on the infectious and parasitic agents in marsh deer populations. The occurrence of *Fasciola hepatica* and *Leptospira interrogans* serovar *pyrogenes* is reported for the first time in wild marsh deer from Argentina. Our data will be useful to improve the interpretation of future mortality events. The field implementation of a surveillance network is key to a holistic approach to wildlife diseases.

## Background

Wildlife populations are increasingly threatened by habitat loss and degradation, invasive species, environmental pollution, climate change, and emerging diseases; all these factors are driven by unsustainable natural resource exploitation by humans [1, 2]. In some instances, disease-related mass mortalities have led to local or global species extinctions [2, 3]. The identification of infectious agents in wild species, along with pathogen surveillance in their populations, contributes to the knowledge about ecosystem health and provides valuable information on the eco-epidemiology of infectious diseases [4, 5]. Only few studies have been conducted on wildlife diseases, and especially on their role in species declines, in South America [4]. The establishment of passive surveillance networks in this region could help increase the reports and analyses of wildlife deaths and contribute to a holistic approach to health and conservation.

The marsh deer (*Blastocerus dichotomus*), the largest South American deer, occurs in marshy habitats in east-central and north-eastern Argentina, west-central and southern Brazil, Paraguay, south-eastern Peru, and eastern Bolivia [6]. The marsh deer is generally solitary, although aggregations of up to six animals have been reported during floods [7]. The species is listed as *Vulnerable* by the International Union for the Conservation of Nature (IUCN) [8] and the Red List of mammals of Argentina [9]. Habitat loss and fragmentation due to agricultural development and construction of hydroelectric dams are the major threats to marsh deer [6, 8]. Poaching, dog attacks, and diseases have been recognized as additional threats [6, 8, 9], but their impact has been scarcely studied. In Argentina, habitat reduction and confinement in less productive areas have displaced marsh deer to remote areas of rather challenging access to accomplish field work.

Little is known about potentially pathogenic macro and microorganisms in wild marsh deer. Tick-borne pathogens, such as *Ehrlichia chaffeensis*, *Anaplasma phagocytophilum*, *A. bovis*, *A. marginale*, *A. platys*, *Theileria cervi*, *Babesia bovis*, and *B. bigemina* were detected in marsh deer populations from Brazil [10–13]. Moreover, the ticks *Amblyomma cajennense*, *A. triste*, *Dermacentor nitens*, and *Rhipicephalus microplus* were found infesting populations located in the Brazilian Parana River region [14, 15]. *Haemonchus contortus* was identified in several South American cervid species [16–18] as well as recognized highly pathogenic for marsh deer [19, 20].

In Argentina, marsh deer mortality events were described in Ibera Wetlands [20, 21], but the health status of this population has never been thoroughly evaluated. The exploratory investigation of a winter mortality event in 2007 detected high parasite burdens of *H. contortus* in association with adverse climatic conditions [20]. Furthermore, limited health assessments have been performed on a few marsh deer individuals in this area since the 1990s. *Trichostrongylus* spp., *Trichuris* spp., and *Ascaris* spp. eggs were reported in faecal samples. *Paramphistomum cervi* was found in one marsh deer, and another deer was infected with *R. microplus, Demodex* sp*.*, and *Chorioptes* sp*.* [21]. Recently, our group documented the occurrence of *E. chaffeensis* not only in marsh deer blood samples but also in the ticks infesting succumbed individuals [22].

In this study, we defined a mortality event of *B. dichotomus* as a spatio-temporal cluster of dead deer deemed to be unusual by local members of a participatory surveillance network. The identification of infectious and parasitic agents in visibly sick or dead deer with poor condition during mortality events will guide future studies. Additionally, basic physiological and histological data as well as haematological values from animals with a good body score are important to assess the general health of individuals or populations. They can be used to evaluate the progress of disease and recognize expected values in healthy wild populations as well as those associated with mortality events [23–25].

The inherent difficulties of wildlife health research in remote areas, in combination with the lack of reports on marsh deer health parameters in Argentinian populations, emphasize the importance of opportunistic sampling, as it allows to increase the number, distribution, and variety of samples that can be submitted to a surveillance system. We developed a participatory surveillance network [26] of marsh deer morbidity and mortality in Argentina that not only allowed us to collect essential information to understand future epidemiological scenarios, but also to train participants in early detection of wildlife disease [25, 27].

## Results

### First steps towards building a participatory surveillance network of marsh deer morbidity and mortality

The participatory surveillance network was made up of researchers, field partners (veterinarians, park rangers, livestock and timber producers, and local community), and decision makers in the two largest populations of marsh deer in Argentina located in Ibera Wetlands (IW) and Lower Delta (LD). In IW, the network included personnel from national and provincial protected areas, NGOs, and private veterinarians. In LD, the activities were integrated into the work programme and management structure of the “Marsh Deer Scientific Technical Committee” (CCP) and included samplings during live captures carried out by “Pantano Project”.

**Fig. 1:**
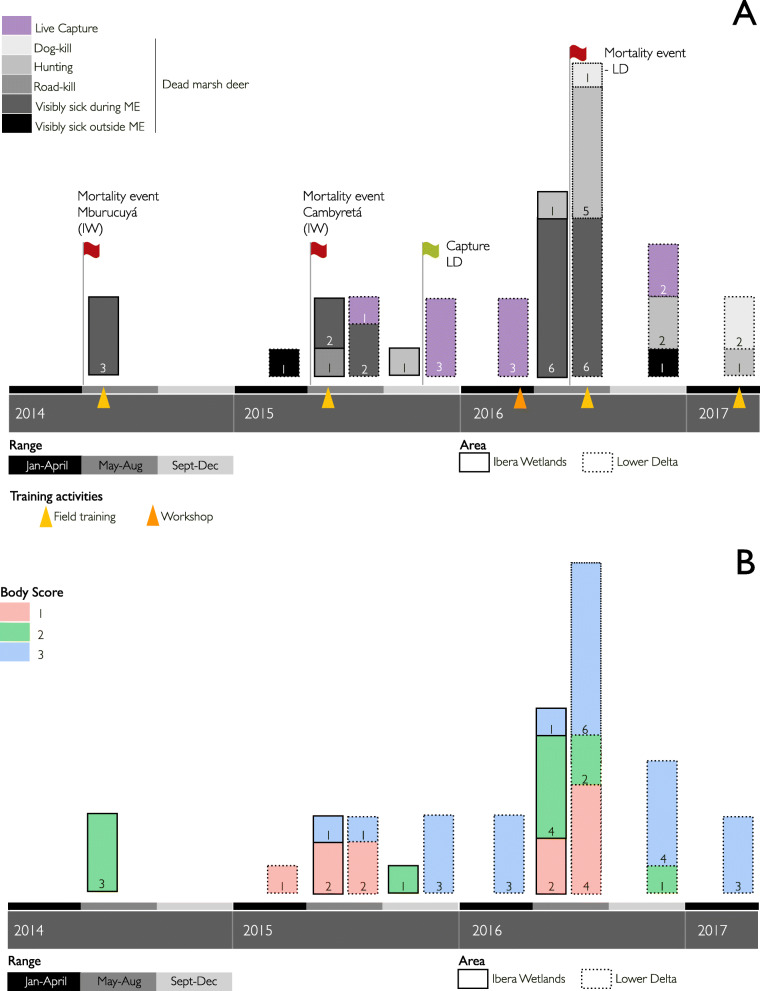
Free-ranging marsh deer sampled in two populations from Argentina between May 2014 and April 2017. Timeline A shows the distribution of marsh deer samplings, cause of death (if applicable), and mortality events reported between 2014 and 2017. Timeline B shows the body score of sampled marsh deer between May 2014 and April 2017. Triangles show the temporal location of training activities. References: ME: Mortality event

The work was gradually articulated in both study areas. Training classes and workshops led to an increased knowledge and improved sampling and biosecurity skills of local partners. During 2014 and 2015, local partners received practical training in the field. In 2016, together with the National Parks Administration, we organized a workshop for the local community in IW. Participants exchanged information on marsh deer and discussed issues, concerns, and approaches in wildlife health, with the purpose of expanding and strengthening the network. The theoretical-practical workshop was attended by 60 persons, including representatives from multiple public, private, and academic institutions and the local community. A similar workshop, held during 2016 in LD, was attended by 15 local partners (Fig. 1a).

### Mortality events

With the support of Provincial Wildlife Agencies and the National Parks Administration, reports of mortality events of marsh deer within the framework of the network took place in May 2014 and June 2015 (Fig. 1a). A total of 27 and 100 carcasses of marsh deer were reported in the Mburucuyá National Park (western IW) and in Cambyretá Area - Ibera National Park (north-eastern IW), respectively. Although most of the dead animals were registered by park rangers during field monitoring in remote areas, a few marsh deer with apparent signs of weakness were observed in the days prior to their death. Necropsies of three marsh deer were performed by our group at each site; the remaining individuals could not be evaluated due to advanced autolysis. During the winter of 2016, trained field partners registered only seven dead animals in IW. A complete necropsy was performed by local partners (veterinarians and park rangers) on two of them. In LD, an extraordinary flood between April and August 2016 coincided with wildlife mortality due to disease and starvation, but some individuals died because they were hit by vehicles, attacked by dogs, or hunted due to increased visibility of animals grouped in the few remaining high and dry areas [28]. Several cases of dead, injured, and hunted marsh deer (*n* = 233) were reported to the CCP [28]. Complete necropsies were performed on nine animals, two of which were found dead with visible signs of disease, four had been sighted sick in the days prior to their death, while the remaining three had been killed by poachers.

In both areas, local partners also reported dead deer that had been hunted, hit by vehicles o killed by dogs outside mortality events; they were sampled opportunistically.

### Marsh deer survey

**Fig. 2:**
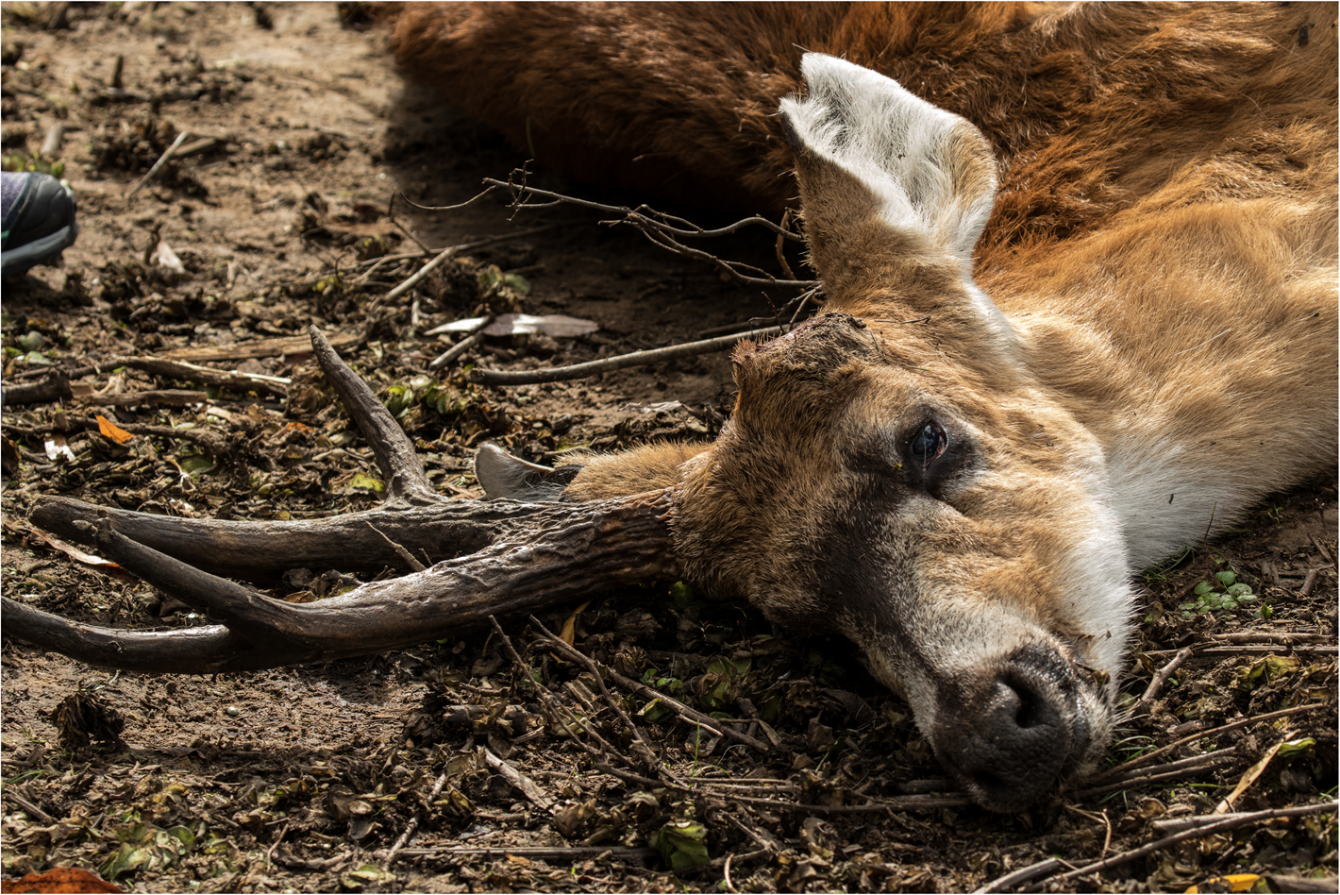
Dead marsh deer sampled during a mortality event (Lower Delta, 2016). Photograph courtesy of Pablo Rodriguez

A total of 44 marsh deer, mostly adult males, were analysed between May 2014 and April 2017. Thirty-five dead animals were sampled in both areas, 19 of which were clinically sick individuals sampled during mortality events (Fig. 2). Live marsh deer (*n* = 9) and the other 16 dead animals, including 10 hunted deer, one road-kill, three dog-kills and two visibly sick individuals, were sampled outside of mortality events. Some deer sampled during and outside mortality events (*n* = 17) had been seen alive before their death (Table 1, Fig. 1a).

**Table 1:** Overview of general information about sampled marsh deer (*Blastocerus dichotomus*) in Argentina.* includes 9 live marsh deer

		Marsh deer sampled		
		During Mortality events	Outside Mortality events	Total
Area	Ibera Wetlands	11	3	14
	Lower Delta	8	22*	30
Age class	Adult	12	13	25
	Yearling	6	12	18
	Fawn	1	0	1
Sex	Male	13	14	27
	Female	6	11	17
Body Condition Score	Score 1	10	1	11
	Score 2	9	2	11
	Score 3	0	22	22
Submandibular oedema	Presence	8	1	9
Cachexia	Presence	10	2	12
Bone fractures	Presence	0	9	9
Skin laceration	Myiasis, alopecia, erosions	11	16	27
Diarrhoea	Presence	9	1	10
Ticks load	Zero - Low	1	17	18
	Medium	6	4	10
	High	8	1	9
	Not evaluated	4	3	7
Pregnancy (n = 17, only females)	Presence	3	2	5
Antler drop (*n* = 26, only adult and yearling males)	Presence	4	1	5

Half of the studied individuals (*n* = 22) had a good body condition (score 3). The other half showed a regular (score 2) or a poor body condition (score 1), and most of them were sampled during mortality events (Table 1). All marsh deer with body score 1 showed cachexia and skin laceration. Submandibular oedema and diarrhoea were frequent in individuals with regular or poor body condition score. During winter, five females were pregnant at time of death, in correspondence with the reproductive season. Five adult or juvenile males had lost their antlers (Table 1). The temporal distribution of sampling, body scores, and cause of death (if applicable) of marsh deer are detailed in Fig. 1a and b.

### Haematology and biochemistry values

Haematological and serum biochemistry parameters of marsh deer were determined in animals sampled alive and with good body condition. In addition to the nine live animals, we included three individuals that had been rescued with gunshot wounds and were sampled while alive, and then died (Table 2). Almost all of the mean haematology values were within the range previously reported for free-living marsh deer in Brazil [29]. Significant differences with the values reported by Szabó et al. [29] were observed in the packed cell volume for both sexes; red blood cell count for both sexes; mean cell haemoglobin concentration in females; and total protein in both sexes [29] (Supplementary material, Additional files 1, 2).

**Table 2:** Haematological and serum biochemistry parameters for marsh deer (*Blastocerus dichotomus*) in Argentina. Sample sizes differed between parameters because insufficient blood could be obtained from some animals

Parameter	n	Median	Range
Packed cell volume (%)	12	32	20–39
Red blood cell count (10^6^/μl)	12	6.22	1.09–10.54
White blood cell count (10^3^/μl)	12	6.20	4.05–9.82
Haemoglobin (g/dL)	5	10.9	8.16–15.7
Mean cell volume (fl)	4	41.43	32.25–43.2
Mean cell haemoglobin (%)	4	15.68	13.15–18.5
Mean cell haemoglobin concentration (g/dL)	4	40.92	33.03–44.5
Total protein (g/dL)	12	6.7	5.8–7.2
Albumin (g/dL)	12	3.16	2.88–3.56
Blood urea nitrogen (mg/dL)	12	39.03	27.81–78.70
Creatinine (mg/dL)	12	1.33	1.00–2.27
Aspartate aminotransferase (IU/L)	12	87.44	23.80–420.02
Alanine transferase (IU/L)	12	20.58	5.74–81.01
Alkaline phosphatase (IU/L)	12	362.53	90.40–1104.60
Creatine phosphokinase (IU/L)	7	203.90	43.60–815.10
Total calcium (mg/dL)	11	7,06	1.55–8.63
Phosphorus (mg/dL)	9	6.29	3.07–8.11
Magnesium (mg/dL)	11	1.94	0.75–4.20

### Serological analyses

The results of the serological analyses are shown in Table 3. Two marsh deer from LD showed evidence of exposure to *Leptospira interrogans* serovar *pyrogenes*. One of them, sampled alive during a CCP rescue, had a good body condition and a *L. pyrogenes pyrogenes* titre of 1/200; the other one, sampled during a mortality event, had a poor body condition and a titre of 1/100. One hunted marsh deer from IW with regular body condition showed evidence of exposure to brucellosis in all four serological techniques (Table 3).

**Table 3:** Serological tests and methods used, and results of tests performed in marsh deer (*Blastocerus dichotomus*) pathogens in Argentina

Pathogen	Test procedure (positive titre)	Number positive/number tested
Bluetongue virus	AGID (1:4)	0/29
Infectious bovine rhinotracheitis (Bovine herpesvirus)	ELISA	0/29
Bovine viral diarrhoea virus	ELISA	0/29
Brucellosis	BPA /ROSEBEN /2ME (1:100) / SAT (1:100)	1/29
Foot-and-mouth disease virus	VIAA	0/29
Johne’s disease (*Mycobacterium avium subsp. paratuberculosis*)	AGID	0/29
*Leptospira interrogans* (11 serovars) ^a^	MAT (1:50)	2/29
Bovine leucosis	AGID	0/29
Q Fever ^b^	Indirect multi-species ELISA	0/29
Chlamydial abortion ^c^	Indirect multi-species ELISA	0/29
Vesicular Stomatitis Virus Indiana and New Jersey Serotype	ELISA-LP (liquid phase)	0/29

References: AGID: agar gel Immunodiffusion; ELISA: enzyme-linked immunodiffusion assay; BPA: buffered plate antigen test; ROSEBEN: rose bengal test; 2ME: 2-mercaptoethanol test; SAT: tube agglutination test; VIAA: virus infection-associated antigen; MAT: microagglutination test. Tests performed at National Service of Agri*-*food health and quality (SENASA)

^a^*Leptospira interrogans* serovars *ballum, castellonis, canicola, grippotyphosa, icterohaemorrhagiae, copenhageni, pomona, pyrogenes, sejroe, wolffi, tarassovi*

^b^ID Screen® Q Fever Indirect ELISA Multi-species kit (ID.vet, France)

^c^ID Screen® *Chlamydophila abortus* Indirect ELISA Multi-species kit (ID.vet, France)

### Identification of ticks and diagnosis of tick-borne agents

All marsh deer from IW were parasitized with the ticks *Rhipicephalus microplus* and/or *Amblyomma triste*, and all ticks collected from individuals in LD were identified as *A. triste*. Tick loads were estimated in 37 marsh deer, including live and dead animals sampled up to eight hours post-mortem. High tick loads were found in nine individuals (24.3%), eight of which were from IW and showed regular or poor body condition. In LD, most marsh deer (74%) had a low tick load (Table 1).

The molecular detection of vector-borne agents (VBA) was performed in 40 marsh deer (Table 4). Regarding piroplasmid parasites, *Babesia* sp. was not detected in any deer, whereas *T. cervi* was found in 21 individuals, 11 of them with regular or poor body condition score. *Ehrlichia chaffeensis* was found in five marsh deer, four of which had a poor body condition score. In both areas, different *Anaplasma* species occurred in marsh deer with good, regular, and poor body condition scores. The identified species of the family Anaplasmataceae included *E. chaffeensis, A. platys, A. odocoilei, A. marginale*, and *Candidatus A. boleense. Trypanosoma theileri* and *T. evansi* occurred in marsh deer with good, regular, and poor body condition scores in both areas. *Rickettsia* sp. was not found in any of the examined individuals.

**Table 4:** Results for PCR identification of vector-borne pathogens in marsh deer (*Blastocerus dichotomus*) samples from Ibera Wetlands (IW) and Lower Delta (LD) populations, by body condition score

	Body Score Condition 1 n positive (%)		Body Score Condition 2 n positive (%)		Body Score Condition 3 n positive (%)		Total n positive (%)	
	IW	LD	IW	LD	IW	LD	IW (*n* = 14)	LD (*n* = 26)
*Babesia* sp*.*	0	0	0	0	0	0	0	0
*Theileria cervi*	4 (28.6%)	2 (7.7%)	4 (28.6%)	1 (3.8%)	0	10 (38.5%)	8 (57.1%)	13 (50.0%)
*Ehrlichia chaffeensis*	3 (21.4%)	1 (3.8%)	0	0	0	1 (3.8%)	3 (21.4%)	2 (7.7%)
*Anaplasma platys*	0	0	1 (7.1%)	0	0	1 (3.8%)	1 (7.1%)	1 (3.8%)
*Anaplasma odocoilei*	0	1 (3.8%)	1 (7.1%)	1 (3.8%)	0	5 (19.2%)	1 (7.1%)	7 (26.9%)
*Candidatus A. boleense*	0	0	0	0	0	1 (3.8%)	0	1 (3.8%)
*Anaplasma marginale*	0	2 (7.7%)	0	0	0	4 (15.4%)	0	6 (23.1%)
*Trypanosoma theileri*	0	2 (7.7%)	1(7.1%)	1 (3.8%)	0	4 (15.4%)	1 (7.1%)	7 (26.9%)
*Trypanosoma evansi*	1 (7.1%)	0	0	0	0	2 (7.7%)	1 (7.1%)	2 (7.7%)
*Rickettsia* sp*.*	0	0	0	0	0	0	0	0

A total of 20 individuals in both areas had co-infections with different VBAs during and outside mortality events. Co-infections involved two VBAs in 13, and three VBAs in six marsh deer. Only one animal with poor body condition sampled during a mortality event in LD was co-infected with four VBAs.

### Quantitative and qualitative *analyses of faeces*

**Fig. 3:**
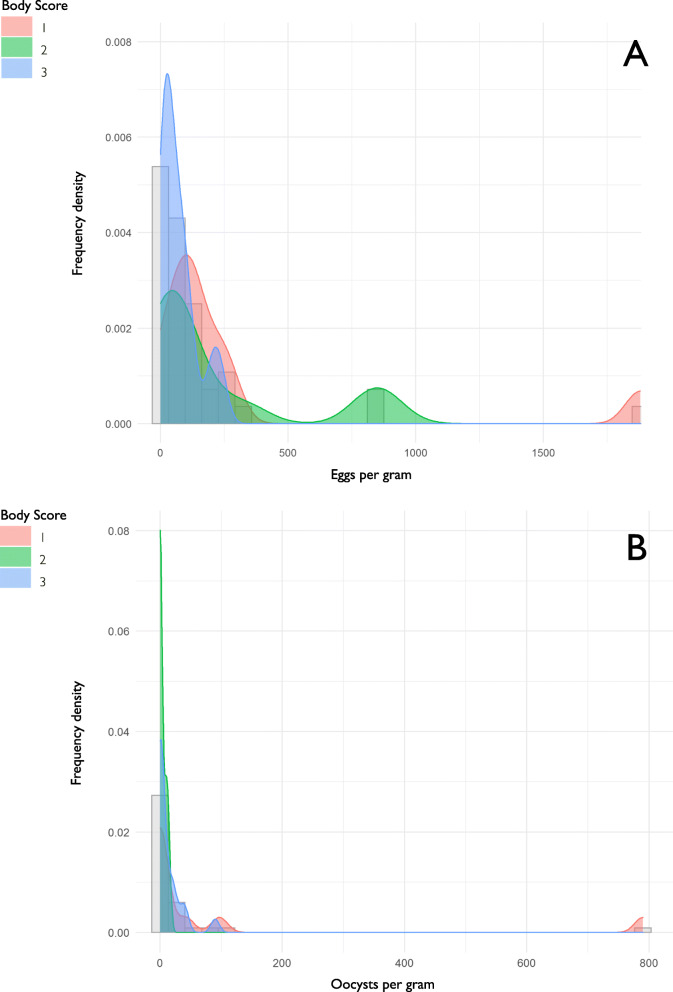
Histogram and relative density distribution of the count per gram of eggs (EPG; **a**) and oocysts (OPG; **b**) in marsh deer (*Blastocerus dichotomus*) from Ibera Wetlands and Lower Paraná Delta, Argentina. The bars represent the relative frequency distribution of EGP and OPG from all animals, and the curves represent the relative density distribution of the EPG categorized by the body score

Faeces from 43 marsh deer were analysed for parasites. More than half of all faecal samples (28/43, 65.1%) had less than 100 parasite eggs per gram (EPG), of which 60.7% (17/28) were from animals with a good body condition. Of the remaining 34.9% (15/43), one marsh deer with poor body condition score had an EPG value of 1880, and two with regular body condition score had EPG values of 826 and 827, respectively. The remaining 12 individuals had EPG values between 100 and 330 (Fig. 3a).

Thirty-one out of 43 animals (72.1%) had less than 10 oocysts per gram (OPG) of faeces. Eleven deer had OPG values between 11 and 100. The highest value (OPG = 790) was found in a dead marsh deer during a mortality event. This animal also had a poor body condition, high tick load, and EPG = 156, including *Trichostrongylus* spp., *Haemonchus* spp., *Ostertagia*, and *Paramphistomum* spp. (Fig. 3b).

Results of the qualitative faecal analyses are shown in Table 5. Trichostrongylina [30], *Strongyloides* spp., *Capillaria* spp., and *Paramphistomum* spp. eggs were found. A high frequency of infection (79%) was detected for Trichostrongylina eggs. Larval culture of 18 faecal samples evidenced the occurrence of *Trichostrongylus* spp. and *Strongyloides* spp., and third-stage larvae morphologically compatible with the genera *Haemonchus*, *Ostertagia*, *Oesophagostomum*, and *Cooperia*. Nine marsh deer were positive for *Haemonchus* sp.; seven of them had a regular or poor body condition and died during mortality events, whereas the three marsh deer positive for *Ostertagia* spp., also sampled during mortality events, had a poor body condition.

**Table 5:** Qualitative analysis of the gastrointestinal parasites identified in faecal samples of marsh deer (*Blastocerus dichotomus*) in Argentina

Parasite	Sugar Flotation	Larval culture
	Number of samples with eggs or oocysts/Total (%)	Number of samples with third stage larvae/Total (%)
Trichostrongylina eggs^a^	34/43 (79%)	–
*Trichostrongylus* spp.	–	12/18 (67%)
*Haemonchus* spp.	–	9/18 (50%)
*Ostertagia* spp.	–	3/18 (17%)
*Cooperia* spp.	–	1/18 (6%)
*Oesophagostomum* spp.	–	1/18 (6%)
*Strongyloides* spp.	13/43 (30%)	5/18 (28%)
*Capillaria* spp.	1/43 (2%)	–
*Paramphistomum* sp.	7/43 (16%)	–
Eimeriidae	22/43 (51%)	–

^a^Ellipsoidal shape, double membrane, smooth surface, medium size (85 μm), with blastomeres according to the different stages

Adult parasites of *Paramphistomum cervi* were identified macroscopically in the rumen of marsh deer during necropsies, and their eggs detected in faeces (Table 5). Six of the seven animals positive for this parasite were from IW; five of them had died during mortality events and showed a poor or regular body condition.

### Gross and histopathological findings

Complete necropsies were performed on 23 marsh deer (14 males and 9 females); 12 of them were adults, 10 juveniles, and one was a fawn. Eight (35%) deer had a good, five (21.5%) a regular, and 10 (43.5%) a poor body condition score. Table 6 summarizes the main microscopic findings in dead animals with score 1 and 2 sampled during mortality events vs. animals with score 3 that died of traumatic causes. One marsh deer with score 2 and sampled outside a mortality event was excluded from Table 6. This animal was reported dead in a field in LD. The deer had inflammation and myiasis in the metacarpophalangeal joint of the left forelimb and a fracture in the second phalanx of the same forelimb.

**Table 6:** Microscopic findings in the main organs examined in dead marsh deer (*Blastocerus dichotomus*) from Argentina

Microscopic findings	Marsh deer (body condition score 1–2) found dead during mortality event	Marsh deer (body condition score 3) dead by trauma
	Number of positive by histopathology /Total	
Heart		
Inflammation	2/13	1/8
Haemorrhages	1/13	0/8
Necrosis	1/13	0/8
Lungs		
Congestion	3/13	4/8
Pneumonia	4/13	3/8
Oedema	2/13	3/8
Haemorrhages	2/13	3/8
Abomasum		
Inflammation	4/10	4/7
Oedema	3/10	0/7
Liver		
Inflammation	7/14	5/8
Hepatocellular steatosis	3/14	0/8
Congestion	3/14	1/8
Necrosis	1/14	0/8
Kidneys		
Inflammation	5/13	2/8
Necrosis	2/13	0/8
Spleen		
Hemosiderosis	4/12	2/8
Lymphoid hyperplasia	2/12	2/8
Lymphoid necrosis	1/12	0/8
Congestion	2/12	0/8
Brain		
Congestion	1/9	1/4
Inflammation	0/9	1/4

Eight of the necropsied marsh deer died of traumatic causes. Three animals had received gunshot wounds to the thorax and were diagnosed with oedema, congestion, and pulmonary haemorrhages (Table 6). Three individuals had been wounded in other parts of the body, and two animals had been attacked by dogs, showing haemorrhages and non-specific lesions in different tissues.

In addition to the necropsied individuals that died of traumatic causes, histopathological analysis allowed us to determine the cause of death of seven animals (two from IW and five from LD) that had poor body condition and died during mortality events. The severe leucocytosis in most organs and multiple histopathological lesions (multifocal hepatic necrosis, lymphoid necrosis of the spleen, and interstitial pneumonia) (Table 6) detected in one marsh deer from IW were compatible with septicaemia. This animal also showed cachexia, submandibular oedema, skin laceration, high tick load, OPG = 790, and was co-infected with *T. cervi, E. chaffeensis, Trichostrongylus* spp., *Haemonchus* spp., *Ostertagia* spp., and *Paramphistomum* sp. The other marsh deer from IW had a severe acute cortical nephrosis; it also showed high tick load and was co-infected with *T. cervi* and *T. theileri.*

One deer from LD had an acute multifocal myocardial necrosis (Table 6). In addition, it showed oedema in multiple organs, a mild nephritis, abomasitis, and EPG = 1880. Cachexia, submandibular oedema, skin laceration, and high tick load were also found, and the deer was co-infected with *T. cervi*, *T. theileri, Trichostrongylus* spp., and *Ostertagia* spp.

Fibrinous bronchopneumonia may have been the cause of death of one deer sampled in 2016 during the mortality event in LD. This animal was co-infected with *T. cervi*, *T. theileri*, and *Trichostrongylus* spp. Another marsh deer sampled in LD died of nephrotoxic nephrosis (Table 6). Clinical conditions and tissue lesions related to malnutrition were detected in this animal and in two other marsh deer sampled in the same area during the mortality event. All three animals were males, showed cachexia, and two of them had lost their antlers days before their death (the third one was a fawn). One deer was co-infected with *T. cervi, A. odocoilei, A. marginale*, and *T. theileri*, and had a hepatocellular atrophy. The fawn had a multifocal pustular rumenitis, atrophy of the thymus, absence of fat reserves, and hepatic lipidosis, and was infected with *E. chaffeensis*.

In the other eight animals, the lesions were non-specific and did not allow us to establish the cause of death. One deer died with a regular body condition score outside the mortality events, while the other eight individuals showed poor or regular body condition and died during mortality events. Six marsh deer showed cachexia, four of which also had submandibular oedema. Five had high, and four intermediate tick loads.

Cysts of *Sarcocystis* sp. were detected in the cardiac muscle of six animals; in two of them, cysts were also found in skeletal muscle. Embryonated eggs and larvae of *Metastrongylus* nematodes were found in seven marsh deer (three juveniles and four adults), whereas adult nematodes were observed only in one adult deer. Adult forms of *F. hepatica* were present in the liver of five animals from IW (22%). Unidentified scattered pyriform microorganisms were observed in erythrocytes in brain vessels of one of these animals, a male with poor body condition.

Skin lesions from two marsh deer were examined. A juvenile male presented a chronic, locally extensive, ulcerative dermatitis with associated panniculitis on the neck. The other lesion was a malignant melanoma in the upper right eyelid of an adult male, characterized by fusiform, anaplastic melanocytes, many of them with intracytoplasmic melanin granules.

### Data analysis

**Fig. 4:**
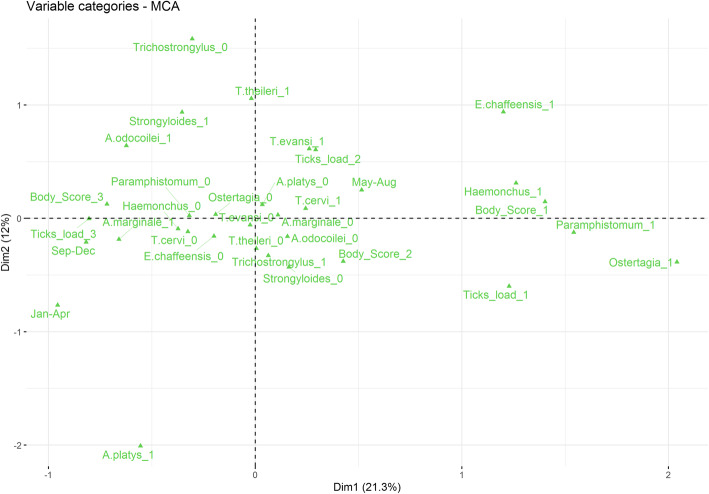
Multiple Correspondence Analysis (MCA) with categorical variables referred to body condition score (Body_score_1, Body_score_2, and Body_score_3); sampling period (Jan-Apr: January to April; May-Aug: May to August; Sep-Dec: September to December); tick load categories (Ticks_load_1: Higher; Ticks_load_2: Medium; Ticks_load_3: Lower); infection with *E. chaffeensis, A. marginale, A. platys*, *A. odocoilei*, *T. cervi*, *T. evansi*, *T. theileri*, and nemathelminthes genera present in faeces: *Trichostrongylus* sp., *Haemonchus* sp., *Paramphistomum* sp., *Strongyloides* sp. and *Ostertagia* sp. The number after the genus or species (0 and 1) refers to negative and positive, respectively

The MCA showed that lower body condition (score = 1), higher tick loads (tick load =1), infection with *E. chaffeensis*, and presence of harmful gastrointestinal parasites (*Ostertagia* sp., *Paramphistomum* sp., and *Haemonchus* sp.) were correlated (Fig. 4). Low body scores and high parasite loads were correlated with the sampling period from May to August (winter season). In the opposite way, the MCA showed that a higher body score was correlated with the September–December sampling period, and with the absence of harmful parasites. These associations were not statistically confirmed.

## Discussion

Along the present study we focused on the development of local networks for the passive surveillance of marsh deer morbidity and mortality in Argentina, which allowed us to reduce the time to detection, reporting, and sampling of visibly sick or dead marsh deer. The networks also allowed us to collect samples from healthy animals during captures and / or rescue procedures. Accurate health parameters in wildlife species are relevant to detect changes in the occurrence of pathogens over time.

The participatory surveillance allowed us to compile information from three mortality events that occurred along 36 months. Although the number of case reports increased, the difficulty of biological sampling in remote areas remains a bottleneck for the comprehensive study of mortality events. Indeed, a larger number of dead animals were found, but no sample collection was possible in many cases because of late detection.

In total we managed to describe the health conditions of 44 marsh deer belonging to the two largest populations in Argentina. We obtained blood parameters from adults with a good body condition, identified the most frequent infectious and parasitic agents in the assessed populations, and characterized tissue lesions from dead animals. Our study revealed the multifactorial source of the mortality events, such as unfavourable environmental conditions, high parasite load, and reduction of body score. The MCA reinforced a possible association between adverse weather conditions during winter periods with lower body score, higher tick loads, infection with *E. chaffeensis*, and presence of well-known harmful gastrointestinal parasites. Larger sample size studies are needed to understand the cascade of factors that trigger mortality events.

Herein we report haematological data of free-ranging marsh deer in Argentina for the first time. Most of the values were in accordance with those published for Brazilian marsh deer populations [29]. To our knowledge, this is the first report of serum chemistry values for this species. These data will be useful for continued health monitoring of marsh deer.

Regarding infectious agents, none of the studied animals showed evidence of exposure to BTV, IBRV, BVDV, foot-and-mouth disease, Johne’s disease, bovine leukosis, Q fever, chlamydial abortion, or VSV. Published information about these infectious agents in cervids, and especially in marsh deer, is relatively scarce. Bovine herpesvirus-1 (BHV-1) causes infectious rhinotracheitis (IBR) in artiodactyls, and a high antibody prevalence was found in marsh deer from Brazil [24]. However, due to their antigenic similarity, BHV-1 cannot be distinguished from cervid herpesvirus-1 and cervid herpesvirus-2 in serological tests [31]. A high seroprevalence (74%) and typical lesions of epizootic haemorrhagic disease virus (EHDV) (an orbivirus related to bluetongue) were detected in marsh deer from Brazil [32], whereas bluetongue virus was reported only in captive *Mazama* sp. In Argentina, serum samples from 14 free-ranging *Ozotocerus bezoarticus celer* were negative to Johne’s disease, BVDV, EHDV, and BTV [33].

It is well-known that human exposure during flood events in Argentina is the most important single risk for leptospirosis [34]. Indeed, a high number of cases of leptospirosis in humans was recorded between 2015 and 2016, coinciding with our sampling period, in the geographical region that includes our sampling site LD [35]. The low number of marsh deer that tested positive for *Leptospira interrogans* during and after a flood event was therefore unexpected. Only two marsh deer from this area were seropositive for the pathogenic *L. interrogans* serovar *pyrogenes*; one of them was found dead without lesions and the other was found alive, with no alterations in haematology and biochemistry values. A single positive titre of 1:100, as found in one of the individuals, can be interpreted as a residual background titre, whereas those between 100 and 200, as found in the second one, are considered relevant in wild herbivores [36]. Although the information on leptospirosis in marsh deer is extremely scarce, studying this pathogen is especially relevant in severe flood scenarios, as its incidence is strongly associated with rainfall and high temperature and humidity levels [37]. Antibodies against *L. interrogans* were found in related species, such as *Mazama gouazoubira* [38] and *O. bezoarticus* (serovars *hardjo*, *mini*, *wolffi*, and *pomona*) [33, 39]. This study documents for the first time antibodies against *L. interrogans* serovar *pyrogenes* in the southernmost marsh deer population in Argentina.

One hunted marsh deer from IW showed evidence of exposure to brucellosis and had relatively high titres. No previous serological analyses in marsh deer from Argentina or Brazil reported evidence of *Brucella* infection [36, 40]. Bovine brucellosis could be an emerging threat for marsh deer, as cattle herds have been detected in the studied area [41].

Although we were unable to estimate the tick load on all marsh deer at the moment of death, the occurrence of high burdens of *A. triste* and *R. microplus* was an important finding in animals with poor body condition, in which skin lesions contributed to an impoverished general condition. This was especially evident during mortality events in IW, where *R. microplus* was the most frequent tick. *Rhipicephalus microplus* mainly infests cattle and is endemic to north-western Argentina, although its geographical distribution does not include LD. The VBAs found in the analysed marsh deer were the same as those observed in Brazil [10–13].

*Ehrlichia chaffeensis*, which causes a zoonotic disease, has recently been described in marsh deer in Argentina [22]. In the present study, its occurrence in animals from IW was positively associated with poor body condition score. Positive deer, except for a fawn, had medium or high tick loads. We suspect that the inflammatory lesions found in the kidney of a positive deer (i.e., glomerulonephritis secondary to immune complex deposition) were caused by *E. chaffeensis*; further immunohistochemical studies might confirm the possible association.

*Theileria cervi* has been historically considered of low pathogenicity, probably because of a long evolutionary relationship between parasite and host [42]. This kind of infection is often asymptomatic in cervids, except for animals with high parasite load, concurrent disease, malnourishment, immunosuppression, in areas with high deer population densities, or in stressful situations [42–44]. Histologically, records of hemosiderosis in tissues of positive marsh deer suggest a possible relationship between the lesions and the agent [45, 46]. In this study, ten out of 11 *T. cervi*-positive deer with poor or regular body condition were assessed during mortality events, and all of them had co-infections with other infectious or parasitic agents. In addition, scattered pyriform microorganisms were found in red blood cells in the brain of one positive deer. Detailed studies of tissues of *T. cervi-*positive deer could support the correlation of infection with specific lesions, especially in populations under stress conditions.

*Trypanosoma theileri, T. evansi, A. platys, A. odocoilei, A. marginale*, and *Candidatus A. boleense* were found in marsh deer from both areas, regardless of their body condition. *Candidatus A. boleense* was first identified in mosquitoes in China [47], and this is the first report in marsh deer in Argentina.

Most of the assessed animals had low EPG and OPG values, and Trichostrongylina eggs, including *Haemonchus* spp., *Ostertagia* spp., and *Trichostrongylus* spp., showed the highest infection frequency. In Argentina and Brazil, helminthic diseases are an important cause of morbidity in this species [16–18], and *H. contortus* was found to be one of the most pathogenic agents involved in mortality events [19, 20, 48]. *Ostertagia* sp. was also described in marsh deer in Brazil [17, 18, 49]. This parasite causes abomasal epithelial hyperplasia and an imbalance in the protein digestion process. During hypobiosis, it can cause petechiae and ecchymotic haemorrhages in the abomasal mucosa [50]. The submandibular oedema found in marsh deer with poor score that died during mortality events could be associated with multiple coinfections with VBAs and harmful gastrointestinal parasites, such as *Haemonchus* spp. and *Ostertagia* spp., as suggested in the exploratory investigation of a winter mortality event in 2007 [20]. The three deer positive for *Ostertagia* spp. were sampled during mortality events, showed co-infection with *Trichostrongylus* spp., and two of them also with *Haemonchus* spp. This record is significant due to the high pathogenicity of these agents in domestic livestock and wildlife [51].

*Paramphistomum cervi* and *Fasciola hepatica* were detected in more than 70% of the necropsied marsh deer from IW. This is the first record of *F. hepatica* in this species in Argentina. The occurrence of *P. cervi* was previously described in animals in Corrientes province, Argentina [21], and high loads of trematodes, including *P. cervi*, were found in individuals from the Parana River basin, Brazil [48]. Both agents had a high prevalence in IW, favoured by suitable ambient temperatures and the simultaneous presence of *Lymnaea* sp. and known definitive hosts (e.g., domestic sheep). In IW, the infection rate in *Lymnaea* sp. increases until the end of summer and autumn, and the clinical signs of disease appear 2–4 months later [52]. Most of the analysed marsh deer had died during mortality events associated with extraordinary floods in IW. The floods increase the area of suitable habitat for *Lymnaea* sp., favouring the production of metacercariae and increasing the infection risk of the definitive host of this trematode. Marsh deer have a higher infection risk due to their habit of feeding in swampy environments. The ecological characteristics of *B. dichotomus*, the detection of liver lesions in some infected individuals, and the high prevalence of *F. hepatica* in the area highlight the importance of further studies to better understand the role of *B. dichotomus* in the maintenance of the infection in the area.

The histopathological findings in succumbed animals allowed us to recognize a broad range of lesions, some of which were associated with body condition. Marsh deer wounded during illegal hunting often flee to swampy areas, where detection is difficult for poachers. Days later, they may appear prowling near human dwellings, where they end up dying. In road-killed or hunted marsh deer, most of the lesions were agonal, such as congestion, oedema, and pulmonary haemorrhages. Other findings were incidental, such as inflammatory reactions in liver, kidneys, lungs, and abomasum. In agreement with the findings described by Navas-Suárez and collaborators [53], they were frequently associated with parasitic agents, such as *F. hepatica*, *Sarcocystis* sp., *Metastrongylus*, or *Trichostrongyloidea* nematodes.

Lesions of the respiratory system were frequent in marsh deer with and without signs of disease. Pneumonia has been described as one of the most frequent inflammatory processes in cervids [53]. In this study, pneumonia was usually mild, except in a juvenile with a locally extended fibrinous bronchopneumonia. Similar to the observations of Navas-Suárez and collaborators [53], lung congestion was the most common hemodynamic disorder, followed by oedema and haemorrhage.

Hepatic inflammations were frequent, but usually not significant or incidental among the examined deer. Digestive tract lesions had a high frequency in marsh deer with poor body condition score. Abomasitis was the most common lesion in the abomasum. It was generally associated with the presence of *Haemonchus* sp. and *Ostertagia* sp., with some animals showing depressions in the abomasal mucosa. A high percentage of intestinal samples showed some degree of autolysis, which hindered bacterial culture and isolation, or a proper histopathological analysis. Some animals had clinical signs of diarrhoea. *Campylobacter* spp. was isolated from faeces of marsh deer in Brazil [54]; in most species, the main clinical sign caused by this bacterium is diarrhoea, which is usually self-limited. Other enteric infections by *Salmonella* spp. and *Yersinia* spp. may affect cervids [36], but they have never been reported in marsh deer.

Only 15 necropsied animals showed lesions that allowed us to determine their cause of death. Eight animals with good body condition died of trauma. Hepatic lipidosis was found in three animals with regular or poor body condition, which could be associated with a negative energy balance during mortality events in the two areas. The nephrosis found in two marsh deer suggested that they died of renal failure, although the cause of the kidney disease could not be established.

In LD, the oedema found in multiple organs of a dead deer with myocardial necrosis may have been associated with heart failure. One individual that died in LD had fibrinous bronchopneumonia, but we were not able to isolate the causal agent due to advanced autolysis at the time of sampling and logistical limitations. The clinical conditions may have been caused by bacteria such as *Mannheimia haemolytica*, *Histophilus somni* or *Pasteurella multocida*, which are frequently detected in stress situations or during co-infections with viral agents [53].

Three dead individuals were sampled in LD in an extraordinary flood during the mortality event of 2016, when food was very scarce. Clinical conditions and tissue lesions of the three dead individuals were related to malnutrition, and one of them had renal failure (see above). The absence of fat reserves and hepatic lipidosis indicated a negative energy balance. The male reproductive cycle and the antler-weakening process are mostly unknown in marsh deer [32]. According to our observations, in both study areas antlers may be shed in almost every month, and older males retain hard antlers for long periods. We were unable to establish whether the loss of antlers in the two adults (the third animal was a fawn) was associated with the antler-weakening process or influenced by malnutrition during mortality events.

With the development and strengthening of the participatory network, we were able to explore marsh deer populations in remote areas, training local collaborators and involving them in the investigation of marsh deer mortalities, a phenomenon they have been witnessing year after year. Through sustained and collaborative work in both areas, we hope to further shorten the time to detection and sampling, thus improving the number and quality of the biological samples. In this study we focused on the agents most commonly mentioned as causing disease in marsh deer and related species, and on those infectious and parasitic agents prevalent in the two study areas. Many of the reported results lay the foundation for future research on the association of infectious agents with pathological lesions in marsh deer, the spillover of agents to and from populations of domestic ruminants, and the role of these cervids in the transmission cycles of certain pathogens. Our work adds important data to the scarce available information on marsh deer health in Argentina [36] and pave the way to future investigations of mortality events.

## Conclusions

The organization and implementation of participatory surveillance networks were the first steps for a comprehensive approach to investigate the morbidity and mortality events of marsh deer in the two largest populations in Argentina. As a first contributions, we provide information on infectious and parasitic agents affecting these populations, and report for the first time the occurrence of *Fasciola hepatica* and *Leptospira interrogans* serovar *pyrogenes* in wild marsh deer from Argentina. Our study reveals the multifactorial source of mortality events, and suggests a possible association of unfavourable environmental conditions with high parasite load and lower body score condition. This experience in participatory surveillance of marsh deer mortality events will be useful to improve wildlife disease monitoring in Argentina.

## Methods

### Study area

Fieldwork was conducted in Ibera Wetlands (IW) and Lower Delta (LD) of Parana River in eastern Argentina. IW is included in a macro-wetland system of 1,300,000 ha (27°40′S, 56°38′W). The region comprises complex ecosystems dominated by paludal environments in Corrientes province. While part of the area is protected (Ibera Provincial Reserve and Ibera National Park), a large portion still consists of private lands. Main productive activities include extensive cattle ranching, agriculture, and tourism [55]. The climate is subtropical with average temperatures of around 15–16 °C, and an absolute minimum temperature of − 2 °C in winter. Mean annual rainfall is around 1500–1800 mm [55]. LD is part of the “Paraná Delta” Biosphere Reserve, located in the Parana River floodplain in Buenos Aires and Entre Ríos provinces (34°15’S, 58°58’W). The area (​​88,724 ha) comprises the typical deltaic morphology with a permanent additional growth of alluvial lands on the outer front of the Parana River. For more than 150 years, LD has been subjected to intensive forestry associated with the construction of dams and roads. At present, subsistence and sport hunting are frequent, although illegal. The climate is temperate, with average temperatures of around 16–18 °C and annual rainfall of 1073 mm [56].

### Establishment of a participatory surveillance network

A surveillance network was developed in each area. Key partners were identified to assist the development of a functional system. Local park rangers and veterinarians were invited to join the participatory surveillance in order to act as focal points to raise awareness and for biological sample collection, respectively. Livestock and timber producers and other members of the local community were trained in marsh deer morbidity and mortality recognition and immediate reporting. Results of the study were shown to decision makers from Provincial Wildlife Agencies and the National Parks Administration, in order to contribute to management strategies.

Methods for training activities ranged from hands-on training for field staff to workshops and informal community gatherings to convey study advances. Workshop presentations were made by epidemiologists, pathologists, and field veterinarians. Appropriate teaching materials and methodologies were developed and used depending on the target audience. The training consisted in data recording, necropsy and sampling techniques, and biosafety and biosecurity standards. The participants were also provided with disposable materials and biosafety kits for sample collection.

At the laboratory, a group of specialists were involved in the diagnostic procedures (histopathological, serological, and molecular diagnosis of infectious and parasitic agents), contributing to the national surveillance system for notifiable diseases. Some samples were sent to the laboratory of the National Service of Agri-Food Health and Quality (SENASA). The information obtained was reported online in the World Animal Health Information System (WAHIS) of the World Organisation for Animal Health (OIE), which summarizes the animal health situation worldwide.

### Marsh deer sampling

Field work consisted of the investigation of animal carcasses or tissues from dead animals during and between mortality events, and was complemented by sampling live animals in one population. Necropsies and sample collection of dead marsh deer were performed by trained local “key partners” or personnel from our group. Free-ranging marsh deer were immobilized by parenteral anaesthesia using tiletamine hydrochloride and zolazepam chlorhydrate (Zelazol®, Fort Dodge) 1.2–1.5 mg/kg combined with xylacine (Xilacina 100®, Richmond Vet Pharma) 0.5–0.8 mg/kg and butorphanol tartrate (Butormin®, Holliday - Scott S.A.) 0.3–0.4 mg/kg [32, 57]. The animals were maintained on thermal padded surfaces and oxygenated through mask (5–8 l/min). Vital signs were continuously measured using a multi-parameter medical monitor (Veterinary Monitor, GT9003C, Fridimex S.A.). The anaesthesia was reversed using naloxone hydrochloride (Naloxona®, Denver Farma) 0.05 mg/kg and yohimbine (Yohimbine Vet®, Richmond Vet Pharma) 0.2 mg/kg.

Live and dead deer were assigned to an age class (fawn, yearling, or mature adult) according to the classical tooth-wear aging technique [58], and body condition (scores 1, 2, 3) was determined using the Body Condition Score Chart [59]. Data about reproductive status, characteristics of the antlers, evidence of submandibular oedema, cachexia, and bone fractures were recorded. All animals were sexed and checked for skin lacerations and presence of ticks by visual inspection and palpation. Ticks were collected manually using acarological tweezers and stored in tubes containing 70° alcohol. External tick load was estimated on each animal using a predefined area of body surface (complete head and neck) and categorized into three levels according to the abundance: category 1 (high load) corresponded to more than 50 ticks, category 2 (medium load) to 30–50 ticks, and category 3 (low / null load) to 0 or less than 30 ticks on the predefined area. Blood samples were collected by jugular vein puncture (10–15 ml, live individuals) or cardiac puncture (15–20 ml, dead individuals). Aliquots of 1 ml each were stored at 4 °C with EDTA, and at − 20 °C and − 80 °C without additives. The remaining blood was centrifuged and serum aliquots were stored at − 80 °C. A faecal sample was collected directly from the rectum of the marsh deer and stored without air in sealable plastic bags at 4 °C.

During necropsies, tissues were macroscopically evaluated and two samples (1 × 1 cm) of selected organs (heart, lungs, abomasum, liver, kidneys, intestine, lymph nodes, spleen, and brain) were collected, including healthy and injured tissue, if present. Tissue samples were fixed in 10% buffered formalin solution (BFS) and frozen at − 80 °C.

### Laboratory diagnosis

The aliquot of blood collected in EDTA tubes was used to determine haematological parameters using manual methods and an automated analyser (Reflotron Plus, Roche, Mannheim, Germany). Blood chemical analyses were performed in an automated analyser (Metrolab 2100, Wiener lab, Rosario, Argentina), and concentration of total protein was determined using a portable refractometer (REF 302, Arcano, China). The blood parameters were compared with previously published data of wild marsh deer from Brazil [29].

Serological diagnoses of bluetongue virus (BTV), infectious bovine rhinotracheitis virus (IBRV), bovine viral diarrhoea virus (BVDV), brucellosis, foot-and-mouth disease, Johne’s disease, *Leptospira interrogans,* bovine leucosis, Q Fever, chlamydial abortion, and vesicular stomatitis virus (VSV) were performed by the National Service of Agri*-*Food Health and Quality (SENASA) according to the procedures described by the World Organization for Animal Health [60].

Ticks were taxonomically identified using a stereoscopic microscope (10X-40X, Nikon SMZ-2 T) and taxonomic keys [61].

DNA was extracted from blood by phenol/chloroform method followed by a standard ethanol precipitation [62]. Blood samples were screened using PCR protocols targeting a fragment of the *16S rRNA* gene for the Anaplasmataceae family [63, 64], a fragment of the internal transcribed spacer *23S–5S* of *Rickettsia* sp. [65], and a fragment of the *18S rRNA* gene for *Trypanosoma* sp. [66] and *Babesia/Theileria* [67]. For positive samples, both strands of the amplified fragment were sequenced with a Big Dye Terminator v3.1 kit from Applied Biosystems and analysed in an ABI 3130XL genetic analyser from the same supplier (Genomic Unit, Consorcio Argentino de Tecnología Genómica (CATG), Instituto de Biotecnología, CICVyA, INTA). Raw files from each gene target were processed using the Vector NTI Advanced 10 program (Invitrogen, Carlsbad, CA, USA). Both chromatograms were used for assembling a consensus sequence. The final file in FASTA format was used for further sequence analysis.

Parasitic elements in faeces were identified, counted, and expressed as EPG (Number of eggs per gram of faeces) or OPG (Number of oocysts per gram of faeces) using a modified Wisconsin technique [68]. Infective larvae were cultured. Taxonomic identification of parasitic elements was performed according to the literature [69–71] using a magnifying glass (10-40X, Carl-Zeiss SV11, Germany).

Tissue samples fixed in 10% BFS were processed using conventional histopathological protocols [72]. Samples were then embedded in paraffin wax and 5 μm sections obtained, which were stained with haematoxylin and eosin. Microscopic lesions found in each tissue were identified and categorized according to severity using a five-degree scale (mild, mild-moderate, moderate, moderate-severe, severe).

### Data analysis

Given the high number of health variables measured, Multiple Correspondence Analysis (MCA) was performed for an exploratory analysis and to visually identify possible associations between body condition and exposure to the different infectious and parasitic agents [73]. MCA is particularly useful in epidemiological studies where large numbers of categorical variables are considered, as it allows to observe clustering of variables without the need to meet data distribution assumptions or a minimum number of cases, as required in other widely used statistical techniques [74, 75].

The variables included were tick load, vector-borne agents (VBA), infection with nemathelminthes species, body score condition, and the period of the year in which the sampling was performed. Given the sensitivity of the MCA for events with low case numbers and even unique cases, only agents with more than one positive individual were included. The t-test was implemented in Epitools [76] to compare the means of the blood values obtained from our work with the means of two references. A significance level of *P* ≤ 0.05 was used (2-tailed).

## Supplementary information

**Additional file 1. **Haematological and serum biochemistry parameters for marsh deer (*Blastocerus dichotomus*) in Argentina.

**Additional file 2. **Haematological parameters by sex, for the marsh deer (*Blastocerus dichotomus*) in Argentina compared with Szabó et al., 2005.

## Data Availability

The datasets used and/or analysed during the current study are available from the corresponding author on reasonable request.

## Abbreviations

2ME: 2-Mercaptoethanol Test
AGID: Agar Gel Immunodiffusion
BFS: Buffered Formalin Solution
BPA: Buffered Plate Antigen Test
BTV: Bluetongue Virus
BVDV: Bovine Viral Diarrhoea Virus
CATG: Consorcio Argentino de Tecnología Genómica
CI: Confidence Interval
CICVyA: Centro de Investigación en Ciencias Veterinarias y Agronómicas
EDTA: Ethylenediaminetetraacetic acid
EHDV: Epizootic Hemorrhagic Disease Virus
ELISA: Enzyme-Linked Immunodiffusion Assay
EPG: Number of Eggs per Gram of faeces
Hg: Haemoglobin
IBRV: Infectious Bovine Rhinotracheitis Virus
INTA: National Agricultural Technology Institute
IU: International Unit
IUCN: International Union for Conservation of Nature
IW: Ibera Wetlands
LD: Lower Delta
MAT: Microagglutination Test
MCA: Multiple Correspondence Analysis
MCH: Mean Cell Haemoglobin
MCHC: Mean Cell Haemoglobin Concentration
MCV: Mean Cell Volume
OPG: Number of Oocysts per Gram of faeces
PCV: Packed Cell Volume
RBC: Red Blood Cell Count
ROSEBEN: Rose Bengal Test
SAT: Standard Agglutination Test
SENASA: National Service of Agri-Food Health and Quality
VBA: Vector Borne Agents
VIAA: Virus Infection-Associated Antigen
VSV: Vesicular Stomatitis Virus
