# Supplementary material for: A participatory surveillance of marsh deer (Blastocerus dichotomus) morbidity and mortality in Argentina: first results
Source: BMC Vet Res. 2020 Sep 1;16:321. doi: 10.1186/s12917-020-02533-x (PMC7465331; doi:10.1186/s12917-020-02533-x)
Supplement: Supplementary file 1 — Additional file 1. Haematological and serum biochemistry parameters for marsh deer (Blastocerus dichotomus) in Argentina. [file 12917_2020_2533_MOESM1_ESM.docx]

**Additional file 1.** Haematological and serum biochemistry parameters for marsh deer (*Blastocerus dichotomus*) in Argentina.

| Parameter | n | Mean | SD |
| --- | --- | --- | --- |
| Packed cell volume (%) | 12 | 30.42 | 6.02 |
| Red blood cell count (10^6^/µl) | 12 | 6.30 | 2.66 |
| White blood cell count (10^3^/µl) | 12 | 6.46 | 1.82 |
| Haemoglobin (g/dL) | 5 | 12.2 | 3.32 |
| Mean cell volume (fl) | 4 | 39.58 | 4.96 |
| Mean cell haemoglobin (%) | 4 | 15.75 | 2.76 |
| Mean cell haemoglobin concentration (g/dL) | 4 | 39.84 | 4.85 |
|  |  |  |  |
| Total protein (g/dL) | 12 | 6.63 | 0.43 |
| Albumin (g/dL) | 12 | 3.21 | 0.24 |
| Blood urea nitrogen (mg/dL) | 12 | 42.89 | 14.06 |
| Creatinine (mg/dL) | 12 | 1.43 | 0.4 |
| Aspartate aminotransferase (IU/L) | 12 | 113.07 | 108.09 |
| Alanine transferase (IU/L) | 12 | 26.52 | 21.05 |
| Alkaline phosphatase (IU/L) | 12 | 397.68 | 299.63 |
| Creatine phosphokinase (IU/L) | 7 | 321.03 | 292.57 |
|  |  |  |  |
| Total calcium (mg/dL) | 11 | 6.79 | 1.93 |
| Phosphorus (mg/dL) | 9 | 5.99 | 1.61 |
| Magnesium (mg/dL) | 11 | 2.35 | 1.07 |
