# Supplementary material for: A participatory surveillance of marsh deer (Blastocerus dichotomus) morbidity and mortality in Argentina: first results
Source: BMC Vet Res. 2020 Sep 1;16:321. doi: 10.1186/s12917-020-02533-x (PMC7465331; doi:10.1186/s12917-020-02533-x)
Supplement: Supplementary file 2 — Additional file 2. Haematological parameters by sex, for the marsh deer (Blastocerus dichotomus) in Argentina compared with Szabó et al., 2005. [file 12917_2020_2533_MOESM2_ESM.docx]

**Additional file 2.** Haematological parameters by sex, for the marsh deer (*Blastocerus dichotomus*) in Argentina compared with Szabó et al., 2005.

| **Male** | **Present study** | | |  |  | **Wild anesthetized (Sbazó et al., 2005)** | | |  |
| --- | --- | --- | --- | --- | --- | --- | --- | --- | --- |
| Parameter | n | Mean | SD |  |  | n | Mean | SD | P-value |
| Packed cell volume (%) * | 8 | 31.25 | 4.4 |  |  | 15 | 39 | 5 | 0.001* |
| Red blood cell count (10^6^/µl) * | 8 | 6.24 | 3.11 |  |  | 15 | 8.3 | 1.6 | 0.046* |
| White blood cell count (10^3^/µl) | 8 | 5.99 | 1.93 |  |  | 15 | 9.90 | 3.60 | 0.996 |
| Haemoglobin (g/dL) | 3 | 12.43 | 2.84 |  |  | 15 | 13.48 | 2.3 | 0.495 |
| Mean cell volume (fl) | 2 | 42.23 | 1,38 |  |  | 15 | 48.4 | 7.2 | 0.258 |
| Mean cell hemoglobin (%) | 2 | 15.68 | 2.91 |  |  |  |  |  |  |
| Mean cell hemoglobin concentration (g/dL) | 2 | 37.05 | 5.69 |  |  | 15 | 34.4 | 3.9 | 0.398 |
| Total protein (g/dL) * | 8 | 6.42 | 0.36 |  |  | 15 | 8.0 | 0.6 | <0.001* |
|  |  |  |  |  |  |  |  |  |  |
|  |  |  |  |  |  |  |  |  |  |
| **Female** | **Present study** | | |  |  | **Wild anesthetized (Sbazó et al., 2005)** | | |  |
| Parameter | n | Mean | SD |  |  | n | Mean | SD | P-value |
| Packed cell volume (%)* | 4 | 28.75 | 9.07 |  |  | 29 | 43 | 4 | <0.001* |
| Red blood cell count (10^6^/µl) * | 4 | 6.42 | 1.79 |  |  | 29 | 9.20 | 1.82 | 0.007* |
| White blood cell count (10^3^/µl) | 4 | 7.37 | 1.33 |  |  | 29 | 9.00 | 2.90 | 0.283 |
| Haemoglobin (g/dL) | 2 | 11.87 | 5.25 |  |  | 29 | 14.7 | 2.25 | 0.12 |
| Mean cell volume (fl) | 2 | 36.92 | 6.61 |  |  | 29 | 47.8 | 8.6 | 0.092 |
| Mean cell hemoglobin (%) | 2 | 15.82 | 3.78 |  |  |  |  |  |  |
| Mean cell hemoglobin concentration (g/dL) * | 2 | 42.63 | 2.64 |  |  | 29 | 34.6 | 5.0 | 0.034* |
| Total protein (g/dL) * | 4 | 7.05 | 0.17 |  |  | 29 | 8.2 | 0,8 | 0.008* |

References: (*) Indicates a significant difference (t-test P≤ 0.05) between the groups: present study vs Szabó et al. 2005.
